# Mesenchymal stem cells for hemorrhagic stroke: status of preclinical and clinical research

**DOI:** 10.1038/s41536-019-0073-8

**Published:** 2019-05-13

**Authors:** Marion T. Turnbull, Abba C. Zubair, James F. Meschia, William D. Freeman

**Affiliations:** 1 Department of Neuroscience, Mayo Clinic Florida, Jacksonville, FL USA; 2 Department of Laboratory Medicine and Pathology, Mayo Clinic Florida, Jacksonville, FL USA; 3 Department of Neurology, Mayo Clinic Florida, Jacksonville, FL USA; 4 Department of Neurologic Surgery, Mayo Clinic Florida, Jacksonville, FL USA; 5 Department of Critical Care Medicine, Mayo Clinic Florida, Jacksonville, FL USA

**Keywords:** Stroke, Mesenchymal stem cells

## Abstract

Significant progress has been made during the past few decades in stem cell therapy research for various diseases and injury states; however this has not been overwhelmingly translated into approved therapies, despite much public attention and the rise in unregulated ‘regenerative clinics’. In the last decade, preclinical research focusing on mesenchymal stem/stromal cell (MSC) therapy in experimental animal models of hemorrhagic stroke has gained momentum and has led to the development of a small number of human trials. Here we review the current studies focusing on MSC therapy for hemorrhagic stroke in an effort to summarize the status of preclinical and clinical research. Preliminary evidence indicates that MSCs are both safe and tolerable in patients, however future randomized controlled trials are required to translate the promising preclinical research into an effective therapy for hopeful patients.

## Introduction

The exuberant public demand for stem cells has led to a rise in unregulated ‘regenerative clinics’ around the world offering unproven stem cell therapy of unknown quality and source for hundreds of diseases and conditions.^1^ However, as illustrated by the recent approval in Europe of Alofisel (Takeda),^2^ we are beginning to see emergence of pharmaceutical grade stem cell therapies. Properly controlled studies are ongoing to determine if stem cell therapy is a viable treatment option for many diseases and injury states. This review is focused on the status of preclinical rodent studies and clinical trials of mesenchymal stem/stromal cell (MSC) therapy for hemorrhagic stroke.

Hemorrhagic strokes account for 15% of all strokes, but are responsible for a disproportionate 40% of stroke-related deaths.^3,4^ Moreover, up to 50% of stroke patients are still dependent on care 1 year after initial ictus and report impairments in memory, speech, and daily activities.^5^ Hemorrhagic stroke is caused by blood vessel rupture and subsequent extravasation of blood into the cranium, and can be further divided into subtypes based on the location of the bleed, including subarachnoid hemorrhage (SAH),^6^ intracerebral hemorrhage (ICH), and intraventricular hemorrhage (IVH). Bleeding into the brain results in oxygen and glucose deprivation to perilesional tissue and initiates a secondary inflammatory response that contributes to lesion expansion, is detrimental to patient outcomes, and for which there is a dearth of therapeutics.^7,8^ Surgical therapies focused on acute hematoma evacuation continue to evolve, but their indication remains exceptional,^9,10^ whereas therapies targeted at inhibiting the secondary inflammatory cascade represent an important opportunity to improve patient survival, reduce functional disability, and offer hope to millions of patients worldwide.

MSCs have been extensively investigated as a treatment for ischemic stroke; however they have been less well studied for hemorrhagic stroke.^11–13^ Nonetheless, more than 10 years of preclinical research investigating MSC therapy for hemorrhagic stroke exist and demonstrate functional improvements in a range of animal models of the disease. Therapeutic use of MSCs may repair or regenerate damaged neuronal cells and may reduce secondary neuroinflammatory cascades, which could improve patient outcomes. The first step towards translation from preclinical data to human trials is to build consensus around the safety and tolerability of MSCs to guide future research protocols and coordinate appropriate trial conditions. We may be at the cusp of overcoming these hurdles for hemorrhagic stroke, exemplified by several publications investigating MSCs therapy for hemorrhagic stroke in humans, and the listing of the first Phase I clinical trial for MSC therapy in hemorrhagic stroke in the United States. This review will focus on preclinical and clinical studies that have investigated MSCs for treatment of hemorrhagic stroke.

MSCs are multipotent stromal progenitor cells and the common precursors of bone, adipose, and cartilage tissue. They retain the ability to differentiate into these tissues, and possibly trans-differentiate into cells of other lineages such as neurons and glia.^14,15^ They are derived from easily accessible sources such as bone marrow, adipose tissue, umbilical cord tissue and the placenta, which make them appealing for therapeutics;^16^ however, despite sharing a common name, MSC properties and functions can vary depending on their source of origin. For example, human placenta-derived MSCs have been reported to have a higher expansion and engraftment capacity than bone marrow-derived MSCs (BM-MSCs).^17,18^ Similarly, umbilical cord-derived (UC)-MSCs and adipose tissue-derived MSCs (AT-MSCs) have a higher proliferative capacity than BM-MSCs in vitro.^19,20^ Differences in epigenetics,^21^ transcript expression,^22^ in vivo engraftment,^21^ cell surface expression,^23^ and cytokine secretion^20^ have also been reported among MSCs of different origins. In addition, significant donor-to-donor variability has been reported.^20^

Unlike traditional drug therapies, MSC pharmacology once delivered into the body cannot be measured through customary pharmacokinetic/pharmacodynamic studies, thus elucidation of cell fate after MSC therapy is essential. Studies have demonstrated that within seconds the majority of intravenous administered MSCs are trapped within the lungs of rodent models,^24,25^ however, MSCs also have the ability to ‘home in’ on the site of injury.^26,27^ The exact mechanisms of this trafficking are still unknown, however expression of receptors and adhesion molecules such as chemokines and matrix metalloproteinases are likely involved in this cell migration.^28^ Our understanding of these mechanisms is further complicated by variability in sourcing, culturing, and delivering MSCs.^29^ Nonetheless, it is postulated that MSCs can mediate multiple mechanisms of action, which could make them ideal for the treatment of a wide range of degenerative and inflammatory diseases.

### Preclinical research

**Fig. 1:**
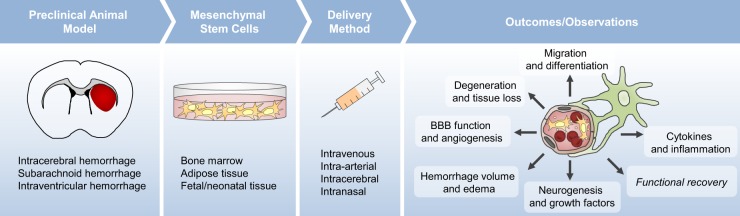
Roadmap of preclinical studies

Investigation into MSC therapy for animal models of hemorrhagic stroke (Table 1; Fig. 1) has been performed for over ten years. Just over half of these studies used MSCs of human origin to treat intracranial hemorrhage, and the rest were sourced from rats. Around 60% of MSCs were sourced from bone marrow (BM-MSCs), as this is a viable source from both humans and rats, whereas umbilical/placental/amniotic-derived cells were used in about one quarter of the studies, and the rest derived from adipose tissue (AT-MSCs). The latter sources were all derived from human tissue. MSCs were generally characterized by expression of cell surface markers assessed through flow cytometry or immunohistochemical methods. MSCs were positive for CD29, CD44, CD73, CD90, and CD105 among others, and negative for the hematopoietic lineage markers, CD14, CD34, and CD45, the stem cell marker CD133, and the marker for endothelial cells, CD144,^30–41^ which is consistent with guidelines.^42^ The delivery method for MSCs also varied, with under half of studies using stereotactically guided intracerebral injection, followed closely by intravenous administration, then intra-arterial and intranasal administration. Although the number of MSCs per dose ranged widely from 1 × 10^5^ to 8 × 10^6^ cells, they tended to split into two groups depending on the delivery method. MSCs delivered via intracerebral injection were given at an average dose of 6.4 × 10^5^ cells per rat (range, 1 × 10^5^ to 5 × 10^6^ cells), whereas MSCs administered intravenously were given at an average dose that was four-fold higher at 2.6 × 10^6^ cells per rat (range, 1 × 10^6^ to 8 × 10^6^ cells). Most studies administered MSCs within one day of injury, followed by between one day and one week after injury. Only one study assessed the efficacy of MSCs for the treatment of chronic stroke and administered MSCs two months after lesion to positive results.^38^

**Table 1:** Preclinical studies of MSC therapy for hemorrhagic stroke

MSC source	Species	Stroke model	Dose, administration, and timing	Results	Ref
Human, bone marrow	Male Wistar rats (270–320 g)	100 µl autologous whole blood into right striatum	3 × 10^6^, 5 × 10^6^ and 8 × 10^6^ cells, by tail vein injection, one day post-ICH	Improvement in NSS; reduced striatal tissue loss; presence of newly formed immature neurons	58
Rat, bone marrow	Sprague-Dawley rats (270–300 g), unknown sex	Collagenase type VII into left caudate nucleus	2 × 10^6^ cells, by carotid artery/ cervical vein/ lateral ventricle injection, on days 1, 3, 5 and 7 after ICH	Improved limb motor function	30
Human, adipose	Male Sprague-Dawley rats (200–220 g)	Collagenase type VII into striatum	3 × 10^6^ cells, by IV injection, 24 h post-ICH	Improvement in modified limb placing behavioral scores; reduced brain atrophy and glial proliferation; endothelial marker expression but not neuronal or glial markers; acute brain inflammation markers	64
Human, processed lipoaspirate (or adipose-derived)	Male Wistar rats (422 ± 28.9 g)	Collagenase type IV into caudate nucleus	3 × 10^6^ cells, by tail vein injection, 24 h post-ICH	Improvement in Rotarod test; no lesion size difference; increase in endogenous progenitor cells	31
Human, bone marrow	Male Wistar rats (270–320 g)	100 µl autologous whole blood into right striatum	1 × 10^6^ cells, by internal carotid artery injection, 24 h post-ICH	No improvement in NSS and corner turn tests from MSC therapy alone (only in combination with mannitol); no striatal tissue loss; presence of newly formed immature neurons	32
Human, umbilical cord	Male Sprague-Dawley rats (230–260 g)	Collagenase type VII into striatum	2 × 10^5^ cells, by intracerebral injection, 24 h post-ICH	Improvement in mNSS and Morris water maze test; injury area significantly reduced; vascular density increased; reduced number of degenerating neurons in peri-ICH area; attenuated immune response	33
Human, umbilical cord *with gene transduction of fibroblast growth factor and hepatocyte growth factor (HGF)	Male Sprague-Dawley rats (mean of 220 g)	Collagenase into internal capsule	6 × 10^5^ cells, by intracerebral injection, one week post-ICH	Improvement in Rotarod test; reduced demyelination	34
Human, bone marrow	Male macaca fascicularis monkeys (4.2 ± 0.2 kg) *first in primate	1.5 mL of autologous arterial blood between the right cortex and basal ganglia	(1–5) x 10^6^ cells, by intracerebral injection, 1 week or 4 weeks post-ICH	Improvement in modified Kito score scale; reduced tissue damage; higher microvessel density	51
Rat, bone marrow (overexpressing GDNF)	Wistar rats (270–320 g), unknown sex	Collagenase type I (0.25 U) and 1 U heparin sodium into right striatum	5 × 10^5^ cells, by intracerebral injection, 3 days post-ICH	Improvement in mNSS; reduced lesion volume	65
Rat, bone marrow	Female Wistar rats (275–300 g)	0.3 mL of blood into the subarachnoid space *first SAH model	3 × 10^6^ cells, by tail vein injection, 24 h post-SAH	Improvement in mNSS; increased numbers of proliferating cells; fewer apoptotic cells	48
Rat, bone marrow	Female Wistar rats (200–250 g)	Collagenase type IV into the striatum	2 × 10^6^ cells, by intracerebral injection, 2 h post-ICH	Enhanced endogenous neurogenesis; reduced apoptosis of newborn neural cells	47
Rat, bone marrow	Male Sprague-Dawley rats (270–320 g)	Collagenase type VII into the striatum	1 × 10^6^ cells, by tail vein injection, one hour post-ICH	Improvement in mNSS; reduced hemorrhage volume; presence of newly formed immature neurons; elevated BDNF	35
Human, adipose	Male Sprague-Dawley rats (200–250 g)	Collagenase type VII into the striatum	1 × 10^6^ cells, by right femoral vein injection, 24 h post-IHC	Improvement in mNSS	36
Human, umbilical cord	Male Sprague-Dawley rats (postnatal day 4 – weight unknown)	200 µL fresh maternal whole blood ventricles (100 µL into each ventricle)	1 × 10^5^ cells, by intracerebral injection, 24 h post-ICH	Prevented PHH development; attenuated impairment on negative geotaxis tests and Rotarod test; reduced corpus callosum loss; increased astrogliosis; increased expression of inflammatory cytokines	59
Human, bone marrow	Male Sprague-Dawley rats (190–210 g)	collagenase type VII into striatum	2 × 10^5^ cells, by intracerebral injection, 1 day post-ICH	Improvement in mNSS; decreased brain water content; reduced neutrophil infiltration and microglial activation in the peri-ICH area; downregulation of inflammatory mediators	37
Rat, bone marrow	Female Wistar rats (200–250 g)	Collagenase type IV (0.5 IU) into striatum	5 × 10^6^ cells, by intracerebral injection, 2 months post-ICH	Improvement in Rotarod and Video-Tracking-Box tests; increased endogenous neurogenesis	38
Rat, bone marrow	Female Wistar rats (275–300 g)	Unheparinized blood into subarachnoid space	3 × 10^6^ cells, by tail vein injection, 24 h post-ICH	Improvement in structural integrity of cerebral tissues (electron microscopy)	68
Rat, bone marrow	Male Sprague-Dawley rats (250–300 g)	Collagenase type IV	3 × 10^6^ cells, by IV injection, two hours post-ICH	Reduced brain edema and blood brain barrier leakage; decreased levels of proinflammatory cytokines; reduced apoptosis; downregulated density of microglia/macrophages and neutrophil infiltration at the ICH site; attenuated ONOO- formation; increased levels of ZO-1 and claudin-5	67
Human, umbilical cord	Male Sprague-Dawley rats (250–300 g)	Collagenase type VII into the striatum	5 × 10^5^ cells, by intracerebral injection, 2 days post-ICH	Improvement in Rotarod tests; reduced lesion volume; increased angiogenesis; reduced inflammatory factors	66
Human, Wharton’s jelly (umbilical cord) *primed for 72 *h into neuron-like cells*	Male Sprague-Dawley rats, (240–280 g)	Collagenase type IV into striatum	2 × 10^5^ cells, by intracerebral injections, 1 week post-ICH	Improvement in Rotarod and limb placing test; increased blood vessel density; increased GDNF in primed cells	43
Rat, bone marrow *with hypoxic preconditioning	Male C57BL/6 mice (25–28 g)	Collagenase type IV	1 × 10^6^ cells, by intranasal delivery, 3 and 7 days post-IHC	Improvement in mNSS, Rotarod test, adhesive removal test, and locomotor function evaluation; reduction in tissue loss; reduction in ventricular enlargement; rescued levels of growth factors; enhanced proliferation and number immature neurons	45
Rat, bone marrow	Male Spontaneously Hypertensive Rat, unknown weight	50 µL of autologous blood	1 × 10^6^ cells, by tail vein injection, timing not recorded	Improvement in mNSS and modified limb placing test; attenuated blood brain barrier permeability; increased levels of tight junction associated protein occludin, and type IV collagen	52
Human, umbilical cord *with hematoma aspiration	Male Sprague-Dawley rats (250–280 g)	Collagenase type IV into caudate nucleus	1 × 10^5^ by intracerebral injection, 6 h post-ICH (with hematoma aspiration)	Improvement in mNSS; reduced p53 expression around hematoma	44
Human, umbilical cord *compares IV and IC administration	Male Sprague-Dawley rats (230–260 g)	Collagenase type VII into the striatum	2 × 10^5^ cells by intracerebral injection. 2 × 10^6^ cells by the tail vein injection - timing not recorded	Improvement in mNSS; reduced injury volume; increased vascular density in intracerebral administration group	46
Human, amniotic membrane	Male Wistar rats (240–260 g)	Collagenase type VII into the striatum	5 × 10^5^ cells, by intracerebral injection, 24 h post-ICH	Improvement in mNSS; increased blood vessel density; reduced apoptosis; increased proliferation and differentiation of neurons; increased growth factor levels; reduced neutrophil infiltration and microglial activation	39
Rat, bone marrow *compared to conditioned media	Male Sprague-Dawley rats (250–280 g)	100 µl autologous arterial blood into right basal ganglia	Dose not reported, by tail vein injection, immediately post-ICH.	Improvements in forelimb-placing, corner turn tests, and mNSS; no effect on Morris water maze performance; reduced brain water content; increased phosphorylation of downstream signaling molecules; decreased inflammatory cytokines	56
Rat, bone marrow *with 2nd messenger signaling inhibitors	Male rats (250–280 g)	100 µl autologous arterial blood into right basal ganglia	Dose not reported, by IV injection, 1 and 24 h post-ICH.	Improvement in mNSS which are blocked by inhibitor treatment; attenuation of second messenger signaling by inhibitors	57
Rat, bone marrow	Male Spontaneously Hypertensive Rats (250–300 g)	20 µl of hemoglobin into right caudate nucleus	1 × 10^6^ cells, by intracerebral injection, 6 h post-ICH	Improvements in mNSS and modified limb placing test; reduced brain water content; reduced apoptosis; increased ZO-1 staining; reduced microglial activation; decreased inflammatory cytokines	40
Human, bone marrow *MSCs to prevent aneurysmal rupture	Male C57BL/6 J mice, weight unknown	Deoxycorticosterone acetate-salt to induce systemic hypertension. Elastase into the right basal cistern	1 × 10^6^ cells, by IV injection, 6 and 9 days after aneurysm induction	Reduced both the incidence of ruptured aneurysms and rupture rate	50
Human, placenta derived	Male Sprague-Dawley rats (250–350 g)	Collagenase type IV into striatum	1 × 10^6^ cells, by tail vein injection, one hour post-ICH	Decreased mortality rate; reduced hematoma volume and ventricular enlargement; reduced brain edema; increased ZO-1 and occludin	41
Rat, bone marrow	Male Wistar rats (300–350 g)	Perforation of the Circle of Willis	1.5 × 10^6^ cells, by intranasal delivery, 6 days post-SAH	Improvement in sensorimotor and mechanosensory function; reduced gray and white matter loss; increased activation of astrocytes and microglia	61

*mNSS* modified neurological severity score, *BDNF* brain-derived neurotrophic factor, *BM* bone marrow, *MSC* mesenchymal stem cell, *GDFN* glial cell-derived neurotrophic factor, *SAH* subarachnoid hemorrhage, *ICH* intracerebral hemorrhage, *IC* intracerebral, *IV* intravenous, *ZO-1* zonula occludens protein-1, *ONOO-* peroxynitrite, *PHH* post-hemorrhagic hydrocephalus

Once administered, the engraftment and differentiation of MSCs into other cell types was assessed. BM-MSCs^30^ and fetal/neonatal tissue derived-MSCs^39,43,44^ were found in the ipsilateral cortex and around the lesion area after intracerebral injection, suggesting that transplanted MSCs are capable of surviving in the perilesional space. Moreover, migration of BM-MSCs to perihematomal sites was observed following intranasal delivery after ICH.^45^ Although there is consensus that migration and survival of MSCs is possible after intracerebral injection of MSCs, there is continued debate on whether MSC migration into the brain is observed with intravenously administrated MSCs.^31,46^

Similarly, groups reported that BM-MSCs,^30,37,38,47,48^ AT-MSCs,^36^ Wharton’s jelly-derived MSCs,^43^ and UC-MSCs^44,46^ were able to differentiate into neurons, astrocytes, and oligodendrocytes in the brain and incorporate into the cerebral vasculature, while others report that only a very small percentage of UC-MSCs differentiate into neurons and glia.^33^ In contrast, Zhou and colleagues report that human amniotic MSCs do not co-localize with any neuronal or astrocyte markers one month after treatment, suggesting that MSCs do not differentiate at all.^39^ Interestingly, AT-MSCs were easily detectable in the spleen up to 28 days after administration,^31^ highlighting the role of the splenic response to stroke.^49^

Most hemorrhagic stroke models used rats; two studies used C57BL/6J mice;^45,50^ and one used *Macaca fascicularis* monkeys (first in primate study).^51^ Sprague-Dawley rats were the most commonly used, followed by Wistar rats, and two separate studies used the spontaneously hypertensive rat (SHR) model, which would seem well-suited for a cerebral hemorrhage model as hypertension is the primary risk factor of human intracerebral hemorrhage.^40,52^ All rat model-based papers investigated MSC treatment across groups of the same sex, with experiments heavily weighted towards male rats, thus it is not possible to reliably assess whether there are sex differences in response to MSC treatment based on animal model data alone. Studies in mouse and primate models were performed exclusively in male animals.^45,50,51^

A number of well characterized experimental models are used to mimic hemorrhagic stroke in animals.^53^ In the studies reviewed, two of the most common methods were employed: direct intracranial injection of whole blood or of bacterial collagenase. A single injection of blood into the intracranial space to mimic hemorrhage has been widely used for almost 40 years,^54,55^ and widely used in the current papers, with autologous blood sourced from the femoral vein or artery.^32,51,52,56–58^ One study also used fresh donor blood, such as maternal blood when 4 day old pups were used.^59^ Injection of collagenase imitates hemorrhagic stroke by disrupting the extracellular matrix and opening the blood–brain barrier (BBB).^60^ Collagenase injection was the most widely used method in the reviewed papers, and similarly to whole blood injection, was administered via direct intracranial stereotactic injection. Only one group perforated the Circle of Willis to induce bleeding, which is more appropriate as a model of human subarachnoid hemorrhage.^61^ Though blood vs collagenase injection methods have been the subject of much debate, neither accurately reproduces all aspects of the human disease. However both protocols result in reproducible hematoma sizes and should continue to be used until better methods are developed.^53,55,62,63^

Changes in sensorimotor and mechanosensory function after MSC therapy were assessed by modified Neurologic Severity Scores (mNSS; a composite of motor, sensory, balance and reflex tests), limb motor function and modified-limb placing tests, corner turn tests, rotor rod performance, negative geotaxis tests (for newborn rats), modified Kito Score (neurological deficit score), adhesive removal test, Video-Tracking-Box test, and locomotor function evaluation. MSC therapy following stroke significantly attenuated impairment in these tests when compared to stroke-only control groups,^30,31,33–40,43–45,48,51,52,56–59,61,64–66^ except for Seyfried and colleagues who report no functional improvements in NSS and corner turn tests when rats were treated with 1 million BM-MSCs, 24 h post-ICH.^32^ In contrast, the same group had previously reported significant improvements in NSS and corner turn tests in rats treated with 3, 5, and 8 million BM-MSCs.^58^ Learning and memory were also tested in rodent models in the Morris water maze paradigm. Liao and colleagues^33^ reported cognitive improvement after UC-MSC therapy with rats, demonstrating reduced latency to the platform compared to the stroke-only groups, which is in contrast to Cui et al.,^56^ who show no change in learning and memory between stroke-only and stroke with BM-MSC therapy groups.

Along with functional outcomes, gross measures of injury such as brain degeneration and lesion size were performed by histological inspection or magnetic resonance imaging (MRI) assessment. Treatment with BM-MSCs,^51,58,61^ AT-MSCs,^64^ UC-MSCs,^34^ and placenta-derived MSCs^41^ after hemorrhagic stroke reduced gray and white matter loss^51,61^ - including reduced striatal tissue loss,^58^ hemispheric atrophy,^64^ and ipsilateral internal capsule loss^34^ - as well as reduced perihematomal glial proliferation,^64^ and decreased stroke-induced ventricular enlargement.^41^ A conflicting report described no difference in striatal tissue volume between fibroblast-treated and BM-MSC-treated groups after stroke; however this was not compared to an ICH only group.^32^ Hemorrhage volumes were also significantly reduced following BM-MSC,^35,65^ UC-MSC,^33,66^ and placenta-derived MSC^41^ therapy compared to stroke only groups, and a comparison of administration methods within the same study found that both intracerebral and intraventricular routes of UC-MSC delivery significantly reduced hematoma volume when compared to stroke alone, but demonstrated no difference in hematoma volume when comparing the two methods of administration.^46^ Only one study using AT-MSCs reported no change in lesion size as assessed by histology and MRI.^31^

Brain edema after BM-MSC^37,40,56,67^ and placenta-derived MSC^41^ treatment was significantly decreased by 1–10% following stroke injury compared to non-treated groups. Moreover, BM-MSC therapy prevented the development of post-hemorrhagic hydrocephalus (PHH) after severe IVH, and reduced compression of the periventricular corpus callosum induced by PHH.^59^ Cerebral tissues, including cerebral arterial walls, were evaluated by electron microscopy, and BM-MSC therapy was found to improve the structural integrity of cerebral tissues,^68^ and attenuate leakage of the BBB.^52,67^ BM-MSC^40,52,67^ and placenta-derived MSC^41^ treatment can also potentially restore BBB disruption through upregulation of BBB integrity proteins, such as claudin-5 and zonula occludens-1 (ZO-1), which are downregulated by stroke, and through suppression of peroxynitrite (ONOO-) formation. Furthermore, BM-MSC,^37,51^ UC-MSC,^33,46,66^ and Wharton’s jelly-derived MSC^43^ treatment, increased perihematomal blood vessel density, suggestive of angiogenesis,^33,37,43,46,51,66^ including a significant increase in von Willebrand factor (an endothelial marker protein)-positive blood vessels.^39^

While the exact mechanisms by which MSCs exert their beneficial effects remain a matter of debate, there are data emerging that MSC-derived exosomes and other secreted factors have the same beneficial effects on hemorrhagic stroke as MSCs.^56,69,70^ Therefore, it is likely that part of the therapeutic action of MSCs is mediated through paracrine secretion of cargo-bearing exosomes, and small molecules such as cytokines. This is exemplified in the current studies, which report that BM-MSC treatment decreased the levels of proinflammatory cytokines interleukin (IL)-1β, IL-2, IL-4, IL-6, tumor necrosis factor (TNF)-α, and interferon (IFN)-γ,^37,40,56,67^ and BM-MSCs^40^ and UC-MSCs^59,66^ increased the levels of anti-inflammatory cytokines IL-10, transforming growth factor (TGF)-β1, IL-1α and IL-1β. These humoral factors can travel throughout the body and affect the biology of both proximal and distant responder cells.^71^ BM-MSCs,^40,61,67^ UC-MSCs,^33,59,66^ AT-MSCs,^64^ and amniotic-derived MSCs,^39^ were also shown to be immunomodulatory as exemplified by reduced astrogliosis,^59,61^ downregulated density of Iba1, CD11b, ED1, CD68, and CD206 immunostained microglia and macrophages,^33,39,40,61,66,67^ and reduced myeloperoxidase (MPO) positive cells, which is representative of neutrophil activation.^33,39,64,66,67^ Moreover, treatment with BM-MSCs,^40,48^ AT-MSCs,^64^ UC-MSCs,^33,59^ and amniotic-derived MSCs^39^ after experimental stroke significantly attenuated the increase in apoptotic and degenerating cells in the perihematomal area.

MSCs have also been shown to promote neurogenesis. This was investigated in a number of the reviewed studies through histochemical staining for markers of proliferating cells, immature neurons, and neuronal precursors. In the perihematomal regions, BM-MSC,^32,47,48,58,61^ AT-MSC,^31^ and amniotic-derived MSC^39^ therapy increased the number of cells positive for these markers two fold, suggesting the presence of newly formed immature neurons. Growth factors also play a role in the therapeutic aspects of MSC function. Bone marrow-^45,56,57^ and amniotic-derived MSC^39^ transplantation rescued the levels of glial cell-derived neurotrophic factor (GDNF), vascular endothelial growth factor (VEGF), and brain-derived neurotrophic factor (BDNF) that were downregulated as a result of experimental stroke,^39,45^ represented by increased phosphorylation of downstream signaling molecules.^56,57^ Moreover, blocking these signaling molecules with specific inhibitors blocked the therapeutic effects of MSCs.^57^

Manipulation of BM-MSCs in vitro prior to use as therapy, such as with hypoxic preconditioning, rescued tissue loss after hemorrhagic stroke injury and reduced the subsequent enlargement of ventricle cavity size.^45^ Similarly, priming of Wharton’s jelly-derived MSCs in vitro with a Rho-associated, coiled-coil containing protein kinase (ROCK) inhibitor increased the expression of GDNF and enhanced their therapeutic potential resulting in improved functional outcomes.^43^ One study combined minimally invasive hematoma aspiration following ICH with UC-MSC treatment and demonstrated that the combination therapy is more effective than either therapy alone,^44^ highlighting the potential of this application in human patients. These studies suggest that using combined approaches may be synergistic.

### Clinical studies

Clinical trials focused on MSC therapy for hemorrhagic stroke are currently limited. A search through *clinicaltrials.gov* comes back with only one result (currently recruiting); while conversely, MSC therapy for ischemic stroke presently lists 13 trials. Despite this underrepresentation in current clinical trials, six research articles have been published of completed trials and case series, ranging from 9 patients to 100, with a total patient count of 164 cases (39.6% female; 106 patients in treatment groups) reported in the literature (Table 2).^72–77^ As with preclinical research, a range of sources was used to obtain MSCs. Bone marrow-derived MSCs,^73,74,77^ and umbilical cord-derived MSCs^72,74,75^ were the most often used in clinical trials. Bone marrow-derived mononuclear cells containing MSCs were also used,^76^ and combination cell transplantation of olfactory ensheathing cells (OEC), neural progenitor cells (NPC), UC-MSCs, and Schwann cells (SCs) were tested.^75^

**Table 2:** Clinical studies of MSC therapy for hemorrhagic stroke

Patient population	Stem cell type	Stroke subtype	Dose, administration, and timing	Follow-up	Functional results and side effects	Ref
12 patients (including 6 controls) 4 females (all MSC group) 8 males, 20–60 years old	Autologous bone marrow-derived MSCs	2 (hemorrhage), 4 (ischemia)	50–60 × 10^6^ cells, via IV administration, 3 months-1 year post-stroke	8 and 24 weeks	No improvement in all clinical scores (FM and BI) and functional imaging parameters at 8 and 24 weeks; no adverse events	73
10 patients, no controls, 6 females, 4 males, 42–87 years old	Combined olfactory ensheathing cells (OEC), neural progenitor cells (NPC), umbilical cord mesenchymal cells (UCMSCs), and Schwann cells (SC)	6 (cerebral infarct), 4 (hemorrhage)	OEC: 1 × 10^6^, OEC + NPC: 1–2 × 10^6^ and 2–4 × 10^6^, NPC: 2–5 × 10^6^, NPC + SC: 2–5 × 10^6^ and 2 × 10^6^, UCMSCs:1–2.3 × 10^7^, via intracranial parenchymal implantation (perilesion) (OEC, NPC), intrathecal implantation (NPC, SC), and intravenous administration (UCMSCs), 6 months-20 years post-stroke	6 months - 2 years	Improvement in neurological function including improved speech, muscle strength, muscular tension, balance, pain, and breathing; increased BI scores and Clinic Neurologic Impairment Scale score; no adverse events	75
100 patients (including 40 controls), 40 females, 60 males, 35–74 years old	Autologous bone marrow mononuclear cells including MSCs	ICH (with surgical drainage and decompressive craniotomy)	7.25 × 10^5^ to 1.35 × 10^6^/L MSCs (3.5mls injected), via intracranial drainage tube (base ganglia), 5–7 days after ICH	6 months	Improvement in NIHSS and BI scores; 5 treatment group patients had low grade fever (3 days) which resolved without intervention; 1 patient was diagnosed with lung cancer 4 months after treatment	76
24 patients (including 8 controls), 8 females, 16 males, 38–58 years old	7 patients with autologous BM mononuclear cells, 9 patients with allograft umbilical cord mononuclear cells	Hemorrhage	1.8 × 10^8^ of BM cells, via intracranial administration into hematoma cavity, 2 weeks then 3 weeks post hemorrhage	3, 6, 12, 36, and 60 months	Computed tomography (CT) scans for brain tissue healing showed better outcomes; improvements in NIHSS, mRS, and modified BI; no adverse events	74
9 patients (including 4 controls), 4 females, 5 males, 41–59 years old	Autologous BM-derived MSCs	ICH	4.57 × 10^7^ MSCs per IV infusion was administered accounting to 8.54 × 10^5^ per kilogram body weight in two occasions (4 weeks apart), > 1 year post ICH	12, 16, 24, 36 and 60 weeks	Improvements in motor disability and cognitive impairment; evident clinical improvement in patients of both groups were comparable; no adverse events	77
9 patients, 3 females, 6 males, 24–30 weeks old	Human umbilical cord-derived MSCs hours	IVH	3 patients received 5 × 10^6^, 6 received 1 × 10^7^, via intraventricular administration within 7 days of diagnosis	2, 4, 6 and 8 weeks	Safe and feasible; no adverse events	72

*BI* Barthel Index, *BM* bone marrow, *FM* Fugl-Meyer, *CT* computed tomography, *ICH* intracerebral hemorrhage, *IV* intravenous, *IVH* intraventricular hemorrhage, *mRS* modified Rankin Scale, *NIHSS* National Institute of Health Stroke Scale, *NPC* neural progenitor cells, *OEC* olfactory ensheathing cells, *SC* Schwann cells, *UCMSCs* umbilical cord mesenchymal stem cells

The first publication for MSC therapy for hemorrhagic stroke was published in 2011 by Bhasin and colleagues.^73^ They used autologous BM-derived MSCs administered intravenously at a dose of 50–60 million cells per patient, and followed up at 8 and 24 weeks. This study included a mix of hemorrhagic and ischemic lesions in the treatment group, and assessed functional recovery and imaging parameters in patients suffering from chronic stroke (3 months to 1 year post-lesion). Despite reporting improvements in functional testing from baseline to follow-up time points post-treatment, these improvements were observed in all groups and were not different between MSC-treated and control-treated patients.

Human studies included neurological impairment and functional assessment measures such as the National Institutes of Health Stroke Scale (NIHSS), the Glasgow Coma Scale (GCS), the Barthel Index (BI), modified Rankin scale (mRS), and the Fugl-Meyer assessment. In the five published cases of MSC treatment for hemorrhagic stroke that measured functional outcomes, four groups reported improvements in these measures relative to the control groups, which is in contrast to the original Bhasin^73^ article, as well as improvements in other measures such as speech, breathing, and pain reporting.^74–77^ Moreover, computed tomography (CT) scans purportedly demonstrate accelerated hematoma reabsorption by 2 weeks after MSC transplantation in patients, however no statistical testing was performed to support this.^74^ These functional effects were reported from 6 months to 5 years after MSC treatment, regardless of MSC source, dose, administration route or timing of treatment. Additionally, in contrast to preclinical rodent studies, human trials were not restricted to a treatment window within a day or week of stroke; instead these six studies were evenly distributed within a continuum of one week to greater than one year post-stroke. This is demonstrated by Tsang and colleagues^77^ who treated patients with severe neurological disabilities one year after onset of ICH. They report improvements in modified BI and functional independence measures 16 weeks post-treatment and an improvement in extended GCS at 60 weeks post treatment when treated with autologous BM-MSCs.^77^

Overall, almost all groups reported a lack of side effects. Patient follow ups for up to 5 years after treatment demonstrate that the therapy is well tolerated, and the trials report almost no adverse events, nor signs of de novo tumor development among patients.^72–75,77^ The exception is Li et al.,^76^ who report that 5 patients (12.5% of their treatment group; compared to one patient (2.5%) in their control group) developed a low-grade fever (38.5 °C), but this resolved within 3 days and without pharmaceutical intervention. This is consistent with a meta-analysis of MSCs in clinical trials which show a significant correlation between MSCs and transient fever,^78^ and could support the idea that MSCs are immune-evasive and not immune-privileged.^79^ Perhaps patient-to-patient variability in immune system function underpins this finding. One patient was diagnosed with lung cancer four months after treatment;^76^ however there is no direct evidence that cell therapy, or MSCs therapy specifically, leads to lung or other cancers.^78,80^ Despite this, treatment with MSCs still warrants further investigation into their long-term safety.

Biomarkers of injury and inflammation were investigated by one group in a Phase I clinical trial of MSC transplantation for severe intraventricular hemorrhage in premature infants. Ahn and colleagues^72^ investigated the temporal profiles of inflammatory cytokines and growth factors in the CSF before and after intraventricular transplantation of umbilical cord blood-derived MSCs. They found reduced levels of the pro-inflammatory cytokine IL-6, but no changes in the levels of TGF-β1, TGF-β2, TNF-α, IL-β, VEGF, fibroblast growth factor (FGF) and BDNF; however this is reported in a premature immune system that might not be representative of an adult immune response.^81^ This is also in contrast to biomarker profiles observed in rodent preclinical research, and highlights the need for further investigation in human patients, or better models for preclinical research.

## Conclusion

Timing, dosage, and route of administration are all variables of an experimental intervention for hemorrhagic stroke that need to be properly considered, controlled for, and, ideally, tested. As stem cells are likely to act as a modulator of the inflammatory response and not as a reducer of ongoing bleeding, delivery is likely optimal beyond the first 24 h when the hematoma has effectively stopped expanding. Dose ranging studies specific to the intervention will need to be done to define ideal dose, which may not be the maximally tolerated dose, and routes of administration to be tested should be feasible in this patient population. As surgery is generally not recommended for hematomal decompression, indirect targeting of the hematomal lesion through intravenous infusion or other non-invasive route would have an appeal. Finally, as fevers are known to worsen neurological outcomes post-stroke, it would be important to closely monitor and, if necessary, mitigate the effects of fever in future trials.

Over 10 years of preclinical research has broadly demonstrated the effectiveness of MSC therapy in experimental hemorrhagic stroke. Moreover, small case studies and series in human hemorrhagic stroke patients have shown improvements in functional recovery with MSC therapy. Given the devastating effects of hemorrhagic stroke, and the millions of patients it affects, there is an understandable drive to develop this therapy for human use. Although a comprehensive understanding of the mechanisms of MSC therapy remains elusive, there is substantial evidence to the effectiveness of these cells as a therapy. A lack of mechanistic clarity has not always been a hurdle for drug development,^82^ even in those as widely used as acetaminophen/paracetamol,^83^ and penicillin.^84^ Initial positive preclinical and clinical results strongly suggest that further investigation into MSC therapy for hemorrhagic stroke is warranted.

### Reporting summary

Further information on research design is available in the Nature Research Reporting Summary linked to this article.

## Supplementary information

Reporting Summary

## Supplementary information

**Supplementary information** accompanies the paper on the *npj Regenerative Medicine* website (10.1038/s41536-019-0073-8).

## References

[1] Sipp D (2017). The malignant niche: safe spaces for toxic stem cell marketing. npj Regen. Med..

[2] European Medicines Agency. *Alofisel*, https://www.ema.europa.eu/en/medicines/human/EPAR/alofisel (2018).

[3] Yang Q (2017). Vital signs: recent trends in stroke death rates-United States, 2000–2015. Mmwr. Morb. Mortal. Wkly. Rep..

[4] Freeman WD, Brott TG (2006). Modern treatment options for intracerebral hemorrhage. Curr. Treat. options Neurol..

[5] Hackett ML, Anderson CS, Group, A. C. R. o. S. H. S. (2000). Health outcomes 1 year after subarachnoid hemorrhage an international population-based study. Neurology.

[6] Burrell C (2016). Precision medicine of aneurysmal subarachnoid hemorrhage, vasospasm and delayed cerebral ischemia. Expert Rev. Neurother..

[7] Jin R, Yang G, Li G (2010). Inflammatory mechanisms in ischemic stroke: role of inflammatory cells. J. Leukoc. Biol..

[8] Mracsko E, Veltkamp R (2014). Neuroinflammation after intracerebral hemorrhage. Front. Cell. Neurosci..

[9] Mendelow AD (2013). Early surgery versus initial conservative treatment in patients with spontaneous supratentorial lobar intracerebral haematomas (STICH II): a randomised trial. Lancet.

[10] Xia Z (2018). Minimally invasive surgery is superior to conventional craniotomy in patients with spontaneous supratentorial intracerebral hemorrhage: a systematic review and meta-analysis. World Neurosurg..

[11] Toyoshima A, Yasuhara T, Date I (2017). Mesenchymal Stem Cell Therapy for Ischemic Stroke. Acta Med. Okayama.

[12] Lucia Maria Ferri A (2016). Mesenchymal stem cells for ischemic stroke: progress and possibilities. Curr. Med. Chem..

[13] Sarmah D (2018). Mesenchymal Stem Cell therapy in Ischemic stroke: a meta‐analysis of preclinical studies. Clin. Pharmacol. Ther..

[14] Pittenger MF (1999). Multilineage potential of adult human mesenchymal stem cells. Science.

[15] Kopen GC, Prockop DJ, Phinney DG (1999). Marrow stromal cells migrate throughout forebrain and cerebellum, and they differentiate into astrocytes after injection into neonatal mouse brains. Proc. Natl Acad. Sci. USA.

[16] Uccelli A, Moretta L, Pistoia V (2008). Mesenchymal stem cells in health and disease. Nat. Rev. Immunol..

[17] Barlow S (2008). Comparison of human placenta-and bone marrow–derived multipotent mesenchymal stem cells. Stem Cells Dev..

[18] Brooke G, Tong H, Levesque JP, Atkinson K (2008). Molecular trafficking mechanisms of multipotent mesenchymal stem cells derived from human bone marrow and placenta. Stem Cells Dev..

[19] Baksh D, Yao R, Tuan RS (2007). Comparison of proliferative and multilineage differentiation potential of human mesenchymal stem cells derived from umbilical cord and bone marrow. Stem Cells.

[20] Russell AL, Lefavor R, Durand N, Glover L, Zubair AC (2018). Modifiers of mesenchymal stem cell quantity and quality. Transfusion.

[21] Reinisch A (2015). Epigenetic and in vivo comparison of diverse MSC sources reveals an endochondral signature for human hematopoietic niche formation. Blood.

[22] Tsai MS (2007). Functional network analysis of the transcriptomes of mesenchymal stem cells derived from amniotic fluid, amniotic membrane, cord blood, and bone marrow. Stem Cells.

[23] Ulrich C (2015). Human placenta-derived CD146-positive mesenchymal stromal cells display a distinct osteogenic differentiation potential. Stem Cells Dev..

[24] Schrepfer, S. et al. In *Transplantation Proceedings*. 573–576 (Elsevier).

[25] Perez JR (2017). Tracking of mesenchymal stem cells with fluorescence endomicroscopy imaging in radiotherapy-induced lung injury. Sci. Rep..

[26] Li M (2016). In vivo human adipose-derived mesenchymal stem cell tracking after intra-articular delivery in a rat osteoarthritis model. Stem Cell Res. Ther..

[27] Drey F (2013). Noninvasive in vivo tracking of mesenchymal stem cells and evaluation of cell therapeutic effects in a murine model using a clinical 3.0 T MRI. Cell Transplant..

[28] De Becker A (2007). Migration of culture-expanded human mesenchymal stem cells through bone marrow endothelium is regulated by matrix metalloproteinase-2 and tissue inhibitor of metalloproteinase-3. Haematologica.

[29] Karp JM, Teo GSL (2009). Mesenchymal stem cell homing: the devil is in the details. Cell Stem Cell.

[30] Zhang H, Huang Z, Xu Y, Zhang S (2006). Differentiation and neurological benefit of the mesenchymal stem cells transplanted into the rat brain following intracerebral hemorrhage. Neurol. Res..

[31] Fatar M (2008). Lipoaspirate-derived adult mesenchymal stem cells improve functional outcome during intracerebral hemorrhage by proliferation of endogenous progenitor cells stem cells in intracerebral hemorrhages. Neurosci. Lett..

[32] Seyfried DM (2008). Mannitol enhances delivery of marrow stromal cells to the brain after experimental intracerebral hemorrhage. Brain Res..

[33] Liao W (2009). Therapeutic benefit of human umbilical cord derived mesenchymal stromal cells in intracerebral hemorrhage rat: implications of anti-inflammation and angiogenesis. Cell. Physiol. Biochem..

[34] Liu AM (2010). Umbilical cord-derived mesenchymal stem cells with forced expression of hepatocyte growth factor enhance remyelination and functional recovery in a rat intracerebral hemorrhage model. Neurosurgery.

[35] Wang SP (2012). Therapeutic effect of mesenchymal stem cells in rats with intracerebral hemorrhage: reduced apoptosis and enhanced neuroprotection. Mol. Med. Rep..

[36] Yang KL (2012). Human adipose-derived stem cells for the treatment of intracerebral hemorrhage in rats via femoral intravenous injection. Cell. Mol. Biol. Lett..

[37] Bao XJ (2013). Transplantation of Flk-1 + human bone marrow-derived mesenchymal stem cells promotes behavioral recovery and anti-inflammatory and angiogenesis effects in an intracerebral hemorrhage rat model. Int. J. Mol. Med..

[38] Vaquero J (2013). Cell therapy with bone marrow stromal cells after intracerebral hemorrhage: impact of platelet-rich plasma scaffolds. Cytotherapy.

[39] Zhou H, Zhang H, Yan Z, Xu R (2016). Transplantation of human amniotic mesenchymal stem cells promotes neurological recovery in an intracerebral hemorrhage rat model. Biochem. Biophys. Res. Commun..

[40] Ding R (2017). Therapeutic benefits of mesenchymal stromal cells in a rat model of hemoglobin-induced hypertensive intracerebral hemorrhage. Mol. Cell.

[41] Choi BY (2018). Human placenta-derived mesenchymal stem cells reduce mortality and hematoma size in a rat intracerebral hemorrhage model in an acute phase. Stem Cells Int..

[42] Dominici M (2006). Minimal criteria for defining multipotent mesenchymal stromal cells. The International Society for Cellular Therapy position statement. Cytotherapy.

[43] Lee HS (2015). Priming Wharton’s jelly-derived mesenchymal stromal/stem cells with ROCK inhibitor improves recovery in an intracerebral hemorrhage model. J. Cell. Biochem..

[44] Zhang Q (2015). Effects of human umbilical cord mesenchymal stem cell transplantation combined with minimally invasive hematoma aspiration on intracerebral hemorrhage in rats. Am. J. Transl. Res..

[45] Sun J (2015). Intranasal delivery of hypoxia-preconditioned bone marrow-derived mesenchymal stem cells enhanced regenerative effects after intracerebral hemorrhagic stroke in mice. Exp. Neurol..

[46] Xie J (2016). Intracerebral and intravenous transplantation represents a favorable approach for application of human umbilical cord mesenchymal stromal cells in intracerebral hemorrhage rats. Med. Sci. Monit..

[47] Otero L (2012). Allogeneic bone marrow stromal cell transplantation after cerebral hemorrhage achieves cell transdifferentiation and modulates endogenous neurogenesis. Cytotherapy.

[48] Khalili MA (2012). Therapeutic benefit of intravenous transplantation of mesenchymal stem cells after experimental subarachnoid hemorrhage in rats. J. Stroke Cerebrovasc. Dis..

[49] Seifert HA, Offner H (2018). The splenic response to stroke: from rodents to stroke subjects. J. Neuroinflamm..

[50] Kuwabara A (2017). Protective effect of mesenchymal stem cells against the development of intracranial aneurysm rupture in mice. Neurosurgery.

[51] Feng M (2011). Serial 18F-FDG PET demonstrates benefit of human mesenchymal stem cells in treatment of intracerebral hematoma: a translational study in a primate model. J. Nucl. Med..

[52] Wang C, Fei Y, Xu C, Zhao Y, Pan Y (2015). Bone marrow mesenchymal stem cells ameliorate neurological deficits and blood-brain barrier dysfunction after intracerebral hemorrhage in spontaneously hypertensive rats. Int. J. Clin. Exp. Pathol..

[53] Strbian D, Durukan A, Tatlisumak T (2008). Rodent models of hemorrhagic stroke. Curr. Pharm. Des..

[54] Ropper AH, Zervas NT (1982). Cerebral blood flow after experimental basal ganglia hemorrhage. Ann. Neurol..

[55] Manaenko Anatol, Chen Hank, Zhang John H., Tang Jiping (2011). Comparison of Different Preclinical Models of Intracerebral Hemorrhage. Intracerebral Hemorrhage Research.

[56] Cui C (2017). Intraparenchymal treatment with bone marrow mesenchymal stem cell-conditioned medium exertsneuroprotection following intracerebral hemorrhage. Mol. Med. Rep..

[57] Cui J (2017). Bone marrow mesenchymal stem cell transplantation increases GAP-43 expression via ERK1/2 and PI3K/Akt pathways in intracerebral hemorrhage. Cell. Physiol. Biochem..

[58] Seyfried D (2006). Effects of intravenous administration of human bone marrow stromal cells after intracerebral hemorrhage in rats. J. Neurosurg..

[59] Ahn SY (2013). Mesenchymal stem cells prevent hydrocephalus after severe intraventricular hemorrhage. Stroke.

[60] Rosenberg GA, Estrada E, Kelley RO, Kornfeld M (1993). Bacterial collagenase disrupts extracellular matrix and opens blood-brain barrier in rat. Neurosci. Lett..

[61] Nijboer CH (2018). Intranasal stem cell treatment as a novel therapy for subarachnoid hemorrhage. Stem Cells Dev..

[62] MacLellan CL (2008). Intracerebral hemorrhage models in rat: comparing collagenase to blood infusion. J. Cereb. Blood Flow. Metab..

[63] MacLellan CL, Silasi G, Auriat AM, Colbourne F (2010). Rodent models of intracerebral hemorrhage. Stroke.

[64] Kim JM (2007). Systemic transplantation of human adipose stem cells attenuated cerebral inflammation and degeneration in a hemorrhagic stroke model. Brain Res..

[65] Yang C (2011). Neuroprotective effects of bone marrow stem cells overexpressing glial cell line-derived neurotrophic factor on rats with intracerebral hemorrhage and neurons exposed to hypoxia/reoxygenation. Neurosurgery.

[66] Kim K (2015). The effect of human umbilical cord blood-derived mesenchymal stem cells in a collagenase-induced intracerebral hemorrhage rat model. Exp. Neurobiol..

[67] Chen M (2015). The inhibitory effect of mesenchymal stem cell on blood-brain barrier disruption following intracerebral hemorrhage in rats: contribution of TSG-6. J. Neuroinflamm..

[68] Khalili MA (2014). Mesenchymal stem cells improved the ultrastructural morphology of cerebral tissues after subarachnoid hemorrhage in rats. Exp. Neurobiol..

[69] Han, Y. et al. Multipotent mesenchymal stromal cell-derived exosomes improve functional recovery after experimental intracerebral hemorrhage in the rat. *J. Neurosurg.* 1–11, 10.3171/2018.2.jns171475 (2018). 10.3171/2018.2.JNS171475 30028267

[70] Otero-Ortega L (2018). Exosomes promote restoration after an experimental animal model of intracerebral hemorrhage. J. Cereb. Blood Flow. Metab..

[71] Wang Y, Chen X, Cao W, Shi Y (2014). Plasticity of mesenchymal stem cells in immunomodulation: pathological and therapeutic implications. Nat. Immunol..

[72] Ahn So Yoon, Chang Yun Sil, Sung Se In, Park Won Soon (2018). Mesenchymal Stem Cells for Severe Intraventricular Hemorrhage in Preterm Infants: Phase I Dose-Escalation Clinical Trial. STEM CELLS Translational Medicine.

[73] Bhasin A (2011). Autologous mesenchymal stem cells in chronic stroke. Cerebrovasc. Dis. extra.

[74] Chang Z (2016). Cell therapy for cerebral hemorrhage: five year follow-up report. Exp. Therap. Med..

[75] Chen L (2013). Multiple cell transplantation based on an intraparenchymal approach for patients with chronic phase stroke. Cell Transplant..

[76] Li ZM (2013). Autologous bone marrow mononuclear cell implantation for intracerebral hemorrhage-a prospective clinical observation. Clin. Neurol. Neurosurg..

[77] Tsang KS (2017). Phase I/II randomized controlled trial of autologous bone marrow-derived mesenchymal stem cell therapy for chronic stroke. World J. Stem Cells.

[78] Lalu MM (2012). Safety of cell therapy with mesenchymal stromal cells (SafeCell): a systematic review and meta-analysis of clinical trials. PLoS ONE.

[79] Ankrum JA, Ong JF, Karp JM (2014). Mesenchymal stem cells: immune evasive, not immune privileged. Nat. Biotechnol..

[80] Sensebé L (2012). Limited acquisition of chromosomal aberrations in human adult mesenchymal stromal cells. Cell Stem Cell.

[81] Simon AK, Hollander GA, McMichael A (2015). Evolution of the immune system in humans from infancy to old age. Proc. R. Soc. B.

[82] Swinney DC, Anthony J (2011). How were new medicines discovered?. Nat. Rev. Drug Discov..

[83] Aronoff DM, Oates JA, Boutaud O (2006). New insights into the mechanism of action of acetaminophen: Its clinical pharmacologic characteristics reflect its inhibition of the two prostaglandin H2 synthases. Clin. Pharm. Ther..

[84] Cho H, Uehara T, Bernhardt TG (2014). Beta-lactam antibiotics induce a lethal malfunctioning of the bacterial cell wall synthesis machinery. Cell.

